# Hospital Construction

**Published:** 1899-01-07

**Authors:** 


### HOSPITAL CONSTRUCTION.

### NEW BUILDINGS AT THE ROYAL PORTSMOUTH HOSPITAL.

The opening of the new buildings at the Portsmouth Royal Hospital, which event, it is hoped, will take place early in the coming year, will mark a great day in the history of the hospital. The present building is a melancholy place, for the most part quite unfitted for a general hospital. It was built and intended for a lock hospital, and two of the wards are still used for their original purpose, in consideration of an annual grant from the Admiralty towards their up-keep, whilst those that have been converted into general wards are sadly lacking in the light and brightness which are associated with modern hospitals.

The new buildings consist of two blocks, with two wards of twenty beds each. Here all the latest improvements in ward construction are to be found. The floors are of terazzo throughout, the corners are rounded, leaving dust as few lurking-places as may be; there are corner windows, that is, one between each end wall and the last bed on each side, as well as between every bed along the whole length of the ward. The patients' lavatories and bathrooms are placed in turrets at the far end of the wards, separated from them by double doors and cross-ventilated lobby; the ward larders and cupboards and lavatories for the nurses are placed right away from the ward, on the main corridor. The larders are tiled in white, and ample ventilation reigns everywhere. In the wards this is secured by ventilators on the floor level, and by fanlight ventilators over each window. The wards are heated with open fires, two stoves in the centre of each ward, the flues being laid beneath the floor.

A small ward for a single bed for specially serious or noisy cases is placed at the entrance to each ward, and opposite is the ward kitchen.

It is now so generally accepted by those best qualified to judge that a sitting-room near her ward is most desirable for a sister, that it seems a great pity a new and fine building should lack this real necessity. But so unfortunately it is at the Portsmouth Hospital; its one drawback from the nursing point of view.

The furnishing and fitting of the wards will soon be in full swing, and it will not be long before a joyful transference of cases will be made to the new buildings, a glad day for all concerned.

